# COVID-19 Vaccine Acceptance in the Democratic Republic of Congo: A Cross-Sectional Survey

**DOI:** 10.3390/vaccines9020153

**Published:** 2021-02-14

**Authors:** John D. Ditekemena, Dalau M. Nkamba, Armand Mutwadi, Hypolite M. Mavoko, Joseph Nelson Siewe Fodjo, Christophe Luhata, Michael Obimpeh, Stijn Van Hees, Jean B. Nachega, Robert Colebunders

**Affiliations:** 1 Kinshasa School of Public Health, University of Kinshasa, Kinshasa 7948, Democratic Republic of the Congo; dalau.nkamba@unikin.ac.cd (D.M.N.); armand.mutwadi@unikin.ac.cd (A.M.); christophe.luhata@pevrdcongo.cd (C.L.); 2 Pôle d’Épidémiologie et Biostatistique, Institut de Recherche Expérimentale et Clinique (IREC), Université Catholique de Louvain (UCLouvain), 1348 Brussels, Belgium; 3 Department of Tropical Medicine, University of Kinshasa, Kinshasa 7948, Democratic Republic of the Congo; hypolite.muhindo@unikin.ac.cd; 4 Global Health Institute, University of Antwerp, 2000 Antwerp, Belgium; josephnelson.siewefodjo@uantwerpen.be (J.N.S.F.); michael.obimpeh@student.uantwerpen.be (M.O.); stijn.vanhees@uantwerpen.be (S.V.H.); robert.colebunders@uantwerpen.be (R.C.); 5 Department of Epidemiology Infectious Diseases and Microbiology and Center for Global Health, University of Pittsburgh, Pittsburgh, PA 15260, USA; jbn16@pitt.edu; 6 Departments of Epidemiology and International Health, Johns Hopkins Bloomberg School of Public Health, Baltimore, MD 21205, USA; 7 Department of Medicine and Center for Infectious Diseases, Stellenbosch University Faculty of Medicine and Health Sciences, Cape Town 7505, South Africa

**Keywords:** COVID-19, vaccine acceptance, determinants and infectious diseases

## Abstract

We investigated the level of willingness for COVID-19 vaccination in the Democratic Republic of Congo (DRC). Data were collected between 24 August 2020 and 8 September 2020 through an online survey. A total of 4131 responses were included; mean age of respondents was 35 years (standard deviation: 11.5); 68.4% were females; 71% had elementary or secondary school education. One fourth (24.1%) were convinced that COVID-19 did not exist. Overall, 2310 (55.9%) indicated they were willing to be vaccinated. In a multivariable regression model, belonging to the middle and high-income category (OR = 1.85, CI: 1.46–2.35 and OR = 2.91, CI: 2.15–3.93, respectively), being tested for COVID-19 (OR = 4.71, CI: 3.62–6.12; *p* < 0.001), COVID-19 community vaccine acceptance (OR = 14.45, CI: 2.91–71.65; *p* = 0.001) and acknowledging the existence of COVID-19 (OR = 6.04, CI: 4.42–8.23; *p* < 0.001) were associated with an increased willingness to be vaccinated. Being a healthcare worker was associated with a decreased willingness for vaccination (OR = 0.46, CI: 0.36–0.58; *p* < 0.001). In conclusion, the current willingness for COVID-19 vaccination among citizens of the DRC is too low to dramatically decrease community transmission. Of great concern is the low intention of immunization among healthcare workers. A large sensitization campaign will be needed to increase COVID-19 vaccine acceptance.

## 1. Introduction

The Coronavirus disease 2019 (COVID-19) burden is still on the rise, especially in Europe and the American continent. As of 31 December 31 2020, the USA had reported over 20.6 million cumulative COVID-19 infections with 351,690 deaths, whereas Africa had reported only 2.8 million cumulative infections and 67,277 deaths, 18,152 and 598 of which were, respectively, from the Democratic Republic of Congo (DRC) [1].

To date, there is no highly efficient treatment for COVID-19, making preventive measures such as mask wearing, hand washing and social distance the only option to curb the pandemic [2,3,4,5,6]. However, the latter have a tremendous impact on the psychosocial well-being of the population [7,8]. At the beginning of November 2020, the first results of large phase-3 COVID-19 vaccine trials were announced [9,10,11]. More than 30 vaccine candidates have been or are currently being assessed through advanced clinical trials [12]. Safety and efficiency data up to 95% were reported [9,10,11]. Several countries around the world have now started to vaccinate people, and many more have started preparations to do so.

If vaccination is well implemented, it could quickly and efficiently reduce the burden of the pandemic [10,11,12,13]. However, a high level of public acceptance and coverage is needed. Therefore, understanding the perceptions of beneficiary communities before investing in immunization is essential, especially in resource-constrained countries [14].

Vaccine hesitancy, defined as any delay in accepting or refusing vaccination despite the availability of vaccine services, is a growing problem around the world [15,16,17]. According to the World Health Organization (WHO), it was one of the public health threats in 2019 and was among the major barriers to achieve the necessary immunization coverage in most of the countries [18]. For example, according to WHO and United Nations International Children’s Emergency Fund (UNICEF) estimations, in 2019, Hepatitis B, Bacillus Calmette Guerin (BCG) and Poliomyelitis (Pol 3) immunization coverage in the Democratic Republic of Congo (DRC) were only 57%, 73% and 59%, respectively [19]. This all underpins the need for good and scientifically reliable information about COVID-19 vaccines and to increase the willingness for COVID-19 vaccination prior to investing in mass vaccination programs.

Little is known about COVID-19 vaccine acceptance and uptake intentions in sub-Saharan Africa. Therefore, in this study we investigated acceptance of a COVID-19 vaccine and its influencing factors in the DRC.

## 2. Materials and Methods

### 2.1. Study Setting and Design

This was a cross-sectional study. An online survey was conducted in the DRC between 24 August 2020 and 8 September 2020. The survey was part of a series of surveys organized by the International Consortium (International Citizen Project COVID-19 (ICPCovid)) and was hosted on the secure web platform of the consortium [6] (www.icpcovid.com). A questionnaire (see full questionnaire in Supplementary Materials 1) proposed by the ICPCovid consortium was translated from English to French and pretested for use in the DRC. The questionnaire included questions on demographic characteristics, educational level and occupation, history of flu-like symptoms and COVID-19 testing, presenting a chronic disease, adherence to preventive COVID-19 measures and willingness to be vaccinated for COVID-19. Part of the results of the survey have been published previously [6]. For this paper, we analyzed two questions of the survey concerning COVID-19 vaccination: (1) Would you consent to receive a COVID-19 vaccine if it becomes available in our country? and (2) If not, why would you refuse the vaccine?

All participants provided e-consent. An URL link to the questionnaire was spread through social media platforms such as WhatsApp and further disseminated through both convenience and snowball sampling, as described previously [6].

### 2.2. Data Processing and Analyses

Completed questionnaires were extracted from the secured server of the ICPCovid website, exported to a Microsoft Excel 2016 spreadsheet for cleaning and coding, and subsequently transferred to STATA version 14.1 (StataCorp, College Station, TX, USA) for analysis.

Community perception about COVID-19 existence was defined as the proportion of respondents in the province who believed that COVID-19 exists. Community COVID-19 vaccine acceptance was defined as the proportion of respondents in the province who would be willing to receive a COVID-19 vaccine. As the two aggregated variables were not normally distributed, we used median values to dichotomize them into high- and low-level categories. Community perception of the existence of COVID-19 was considered high if at least the median of the respondents believed in the existence of the disease. Community COVID-19 vaccine acceptance was considered as high if at least the median of respondents would accept to be vaccinated [20,21].

Categorical variables were summarized as frequencies and proportions, continuous variables as mean and standard deviation (SD) or median and interquartile range (IQR).

The association between dependent and independent variables was determined using crude and adjusted odds ratios (OR), with 95% confidence intervals (95% CI). *P*-values < 0.05 were considered significant. We used two-level mixed-effect logistic regression modelling to simultaneously investigate community and individual level factors associated with the acceptance of a COVID-19 vaccine, considering between-province variation.

Three multilevel logistic regression models were fitted. The first model (Model 1) was an empty model that contained only province-specific random effects to model between-province variation in the acceptance of COVID-19 vaccine. The second model (Model 2) included the province-specific random effects and the respondent characteristics; the third model (Model 3) included province-specific random effects, respondent characteristics and the two province-level characteristics. From the fitted bivariate regression, all variables with a likelihood ratio *p*-value < 0.25 were included in the multivariable analysis. The most parsimonious model was selected throughout a backward stepwise approach, based on the smallest Akaike information criterion (AIC). Multicollinearity among independent variables was checked by estimating the variance inflation factor. All variance inflation factor values were <10, therefore there was no multicollinearity. Variance partition coefficient (VPC) and proportional change in variance (PCV) were used to quantify the magnitude of the effect of province itself on respondent acceptance of vaccine [22].

We evaluated the accuracy of the final model using the receiver operating characteristic (ROC) by measuring the area under the curve (AUC).

### 2.3. Ethical Considerations

The study protocol was approved by the DRC National Ethics Committee (CNES N° 175/CNES/BN/PMMF/17/04/2020). Data were collected online anonymously and were only available to study investigators using passwords.

## 3. Results

### 3.1. Participants’ Characteristics

Overall, 4160 respondents of 17 provinces participated in the survey. Ten provinces were excluded from the analysis because they had fewer than 10 respondents; this excluded 29 respondents. Thus, 4131 participants were included in the analysis from 7 provinces: Haut Katanga, Kasaï Central, Kasaï Oriental, Kinshasa, Congo Central, Kwilu and North Kivu [6]. The mean age was 35 ± 11.5 years; 2827 participants (68.4%) were female, and 2931 (71%) had elementary or secondary school level education. Six hundred and two (14.6%) reported to present a chronic disease; 441 (10.7%) had been tested for COVID-19, of which 35 (7.9%) had a positive result. In total, 996 (24.1%) did not believe COVID-19 existed (Table 1).

### 3.2. COVID-19 Vaccine Acceptance

Overall, 2310 participants (55.9%) reported they were willing to be vaccinated for COVID-19. The highest level of willingness was reported in Kasai Central, where 94% (583/621) of respondents were willing to be vaccinated, followed by Kasai Orientale with 85% (489/578). The lowest willingness was reported in Haut Katanga with 36% (184/511), followed by Kwilu with 39% (269/689) (Table 2).

**Figure 1:**
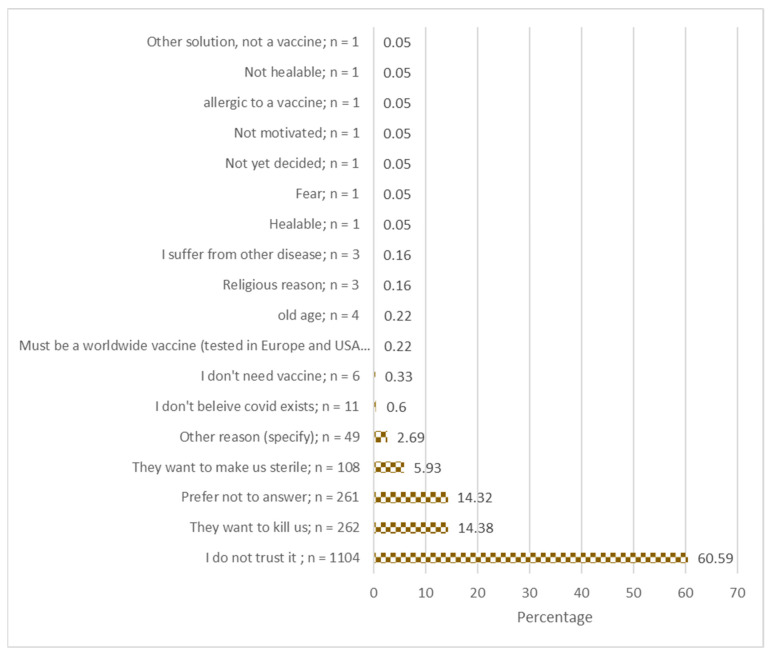
Reasons for COVID-19 vaccine hesitancy; responses of survey participants from seven provinces in the Democratic Republic of the Congo.

Among the 1821 participants not willing to receive a COVID-19 vaccine, the majority (*n* = 1104, 60.6%) indicated they did not trust the vaccine; 262 (14.4%) believed the vaccine is made to kill people in Africa; 108 (5.9%) believed the vaccine is made to sterilize people (Figure 1).

### 3.3. Determinants of COVID-19 Vaccine Acceptance

In multivariate analysis, five variables remained associated with COVID-19 vaccine acceptance. Participants in the middle- or high-income category were more likely to accept COVID-19 vaccination (OR = 1.85, CI: 1.46–2.35 and OR = 2.91, CI: 2.15–3.93, respectively), whereas healthcare workers were less likely to accept a COVID vaccine (OR = 0.46, CI: 0.36–0.58). Respondents already tested for COVID-19 were more likely to accept the vaccine; (OR = 4.71, CI: 3.62–6.12). Respondents who believed COVID-19 exists were more likely to accept the vaccine (OR = 6.04, CI: 4.42–8.23). At the community level, the aggregated willingness was significantly associated with acceptance (OR = 14.45, CI: 2.91–71.65; *p* = 0.001) (Table 3).

Details regarding province-level variance of the multilevel logistic models predicting acceptance of vaccine and the random effect results are included in Table A1.

Community perception of the existence of COVID-19 was considered high if at least 87.3% (median) of the respondents believed in the existence of the disease. Community COVID-19 vaccine acceptance was considered as high if at least 46.9% (median) of respondents would accept to be vaccinated. Also, the ROC showed an area under the curve (AUC) of 82.5%, indicating the model was good enough in differentiating those accepting to be vaccinated for COVID-19 disease from those not accepting to be vaccinated.

## 4. Discussion

We documented an overall low willingness for vaccination among citizens of the DRC. Only about half (56%) of the respondents were willing to receive a COVID-19 vaccine, which is far below what is required to stop the ongoing COVID-19 pandemic. With an R0 of 3, at least 67% ((1–(1/R0) × 100%) of the population is to be vaccinated to stop the pandemic [23,24,25]. To achieve this coverage, a willingness for vaccination of >67% is an absolute prerequisite. With a willingness for vaccination of 56%, it is advisable to prioritize people at risk for severe disease while developing a strategy to improve vaccine willingness in the community. Healthcare workers should also be prioritized, given their high level of exposure to SARS-Cov-2. However, given their low willingness to be vaccinated, this will require a targeted sensitization/education campaign [26,27,28].

About 60% of the responders who were not willing to be vaccinated mentioned that they did not trust the vaccine. This means that strong evidence about vaccine safety and efficacy will be needed to convince communities to accept vaccination.

Already in May 2020, the DRC’s National Vaccination Program conducted a survey to assess barriers to vaccination. In this survey, similar reasons for COVID-19 vaccine hesitancy were identified. Moreover, in the aforementioned survey, data was also collected about sources of rumors and misinformation. Reported sources of information by participants were human-to-human information shared by word of mouth (45%), social media (25%), TV and radio (16%) and other sources, such as traditional practitioners [29]. Although the human-to-human word of mouth channel was cited as the most important way for information about COVID-19, it is difficult to determine the influence of social media in the circulation of rumors and disinformation.

In our study, we identified significant disparity in willingness to be vaccinated between provinces in the DRC. The willingness for vaccination was the highest in central and eastern Kasai, where at least 84% of the respondents were willing to receive a COVID-19 vaccine, which was in contrast with the Haut Katanga and Kwilu, where less than 40% of the respondents were willing to be vaccinated. Though the exact reasons for this disparity remain to be determined, one may speculate that the speed at which rumors and disinformation spread through social media could have played a role. It may be that provinces with low internet and social media penetration are likely to be less exposed to circulating rumors and negative information about the vaccine. However, all respondents who participated in the survey were using WhatsApp and therefore were exposed to messages in social media. The two Kasai provinces have a low coverage of social media and internet [30]. Both are in the center of the DRC and almost landlocked, with little road and air traffic [30]. In contrast, the province of Haut-Katanga, where the willingness for vaccination is very low, is the second province of the DRC in terms of development, very open to the outside with several foreign flights and an open border with Zambia and indirectly with southern Africa, with significant traffic between countries because of mining activities. Similarly, in the capital city of Kinshasa, the willingness to be vaccinated was only 41%.

Studies have shown that large proportions of vaccine information on popular social media sites are antivaccination messages posted by very active vaccine-hesitant groups [31]. Around April 2020, there was a strong reaction in the media in the DRC when it was announced by the head of the Congolese COVID task force that COVID-19 vaccine clinical trials were planned in the DRC. People feared that Congolese people would be used as guinea pigs. To calm the situation, the head of the COVID task force had to explain on TV that only vaccines would be tested that had been tested before in Europe and the United states [32].

We identified several factors that were associated with willingness to be vaccinated. First, belonging to a middle- and high-level income category was associated with an increased willingness for COVID-19 vaccination, possibly due to better access to high quality information, e.g., through better television channels and/or though contact with people living abroad in countries that are severely affected by COVID-19 and/or because such individuals generally live in towns where there is more transmission, hence higher risk to get contaminated. Secondly, participants who had been tested for COVID-19 were more likely to be willing to receive a COVID-19 vaccination. This may also be associated with higher exposure to high quality information and/or a better awareness about the disease and the risks of being infected. Strikingly, being a healthcare worker was associated with decreased willingness for vaccination, which is problematic, as healthcare workers are supposed to provide counselling and to sensitize nonhealthcare workers concerning the COVID-19 vaccine [26,27,28]. Reasons for vaccine hesitancy were quite similar among healthcare workers and other respondents (Table A2). Another study conducted among healthcare workers in the DRC (in March and April 2020) found that only 28% of them would accept to be vaccinated with a COVID vaccine. The authors of this study suggested that this low willingness to be vaccinated was the consequence of the spread of misinformation through social networks. In some high-income countries (Belgium, France, Canada), some COVID vaccine hesitancy among healthcare workers has also been reported because of safety concerns [33].

Several limitations of our study must be acknowledged. First, our respondents are not representative of the general population living in the provinces where the survey was done. Only people with internet connection could participate in the study. As a consequence, 29% of responders had a university degree, 69% belonged to the lower and middle class of the population and 10.8% were healthcare workers or students in the healthcare setting. As the level of education of our responders was higher than that of the general population, we speculate that vaccine hesitancy of the general population may even be more important. Secondly, self-reports may be influenced by recall bias and social desirability.

## 5. Conclusions

We found a low overall willingness for vaccination among citizens in the DRC. Of great concern is that being a healthcare worker was associated with a decreased willingness to be vaccinated. More in-depth research is needed to investigate the reasons for vaccine hesitancy in the different populations and different regions of the DRC. Moreover, vaccine hesitancy will need to be monitored, as it is expected to vary over time, given the speed of certain rumors and misinformation that can be spread by social media. As such, our data call for innovative sensitization/education campaigns to increase the willingness for vaccination before implementing mass-vaccination campaigns. In addition, health authorities should consider cost-effective strategies and criteria, such as age and comorbidities, to prioritize vaccination in different population layers based on the risk related to SARS-Cov-2 infection and COVID-19 mortality [34].

## Figures and Tables

**Table 1:** Participants’ demographic characteristics per province.

Characteristics	Province							Total
	Haut-Katanga	Kasaï-Central	Kasaï-Oriental	Kinshasa	Kongo-Central	Kwilu	Nord-Kivu	
Total	511 (12.4)	621 (15.0)	578 (14.0)	633 (15.3)	616 (14.9)	689 (16.7)	483 (11.7)	4131 (100.0)
**Age *n* (%)**								
≤30	199 (38.9)	75 (12.1)	236 (40.8)	227 (35.9)	336 (54.5)	408 (59.2)	207 (42.9)	1688 (40.9)
31–39	152 (29.7)	325 (52.3)	143 (24.7)	123 (19.4)	138 (22.4)	136 (19.7)	137 (28.4)	1154 (27.9)
40–49	125 (24.5)	164 (26.4)	110 (19.0)	151 (23.9)	87 (14.1)	80 (11.6)	69 (14.3)	786 (19.0)
≥50	35 (6.8)	57 (9.2)	89 (15.4)	132 (20.9)	55 (8.9)	65 (9.4)	70 (14.5)	503 (12.2)
**Gender *n* (%)**								
Male	7 (1.4)	31 (5.0)	137 (23.7)	289 (45.7)	297 (48.2)	337 (48.9)	206 (42.7)	1304 (31.6)
Female	504 (98.6)	590 (95.0)	441 (76.3)	344 (54.3)	319 (51.8)	352 (51.1)	277 (57.3)	2827 (68.4)
**Education *n* (%)**								
Primary/Secondary	475 (93.0)	531 (85.5)	481 (83.2)	429 (67.8)	276 (44.8)	505 (73.3)	234 (48.4)	2931 (71.0)
University	36 (7.0)	90 (14.5)	97 (16.8)	204 (32.2)	340 (55.2)	184 (26.7)	249 (51.6)	1200 (29.0)
**Marital *n* (%)**								
Cohabitation	5 (0.9)	0 (0.0)	157 (27.2)	126 (19.9)	85 (13.8)	72 (10.5)	89 (18.4)	534 (12.9)
Divorced	5 (0.9)	2 (0.3)	11 (1.9)	30 (4.7)	16 (2.6)	1 (0.2)	22 (4.6)	87 (2.1)
Legally married	496 (97.1)	583 (93.9)	374 (64.7)	250 (39.5)	144 (23.4)	190 (27.6)	229 (47.4)	2266 (54.9)
Single	5 (0.9)	30 (4.8)	12 (2.1)	200 (31.6)	364 (59.1)	416 (60.4)	100 (20.7)	1127 (27.3)
Widow/widowed	0 (0.0)	6 (0.9)	24 (4.1)	27 (4.3)	7 (1.1)	10 (1.5)	43 (8.9)	117 (2.8)
**Religion *n* (%)**								
None	2 (0.4)	0 (0.0)	14 (2.4)	32 (5.1)	42 (6.8)	12 (1.7)	18 (3.7)	120 (2.9)
Catholic	281 (55.0)	356 (57.3)	183 (31.7)	170 (26.9)	111 (18.0)	257 (37.3)	195 (40.4)	1553 (37.6)
Protestant	125 (24.5)	14 (2.3)	119 (20.6)	155 (24.5)	127 (20.6)	100 (14.5)	175 (36.2)	815 (19.7)
Revival/Pentecostal	91 (17.8)	16 (2.6)	179 (31.0)	86 (13.6)	27 (4.4)	63(9.1)	22 (4.6)	484 (11.7)
Other	12 (2.3)	235 (37.8)	83 (14.4)	190 (30.0)	309 (50.2)	257(37.3)	73 (15.1)	1159 (28.1)
**Occupation *n* (%)**								
Jobless/Student	301 (58.9)	401 (64.6)	250 (43.2)	296 (46.7)	343 (55.7)	456(66.2)	208 (43.1)	2255 (54.6)
Self-employed	139 (27.2)	156 (25.1)	207 (35.8)	99 (15.6)	59 (9.6)	93(13.5)	159 (32.9)	912 (22.1)
Private sector	36 (7.0)	56 (9.0)	72 (12.5)	90 (14.2)	69 (11.2)	66(9.6)	74 (15.3)	463 (11.2)
Public sector	35 (6.8)	8 (1.3)	49 (8.5)	148 (23.4)	145 (23.5)	74(10.7)	42 (8.7)	501 (12.1)
**Healthcare work *n* (%)**								
No	486 (95.1)	617 (99.4)	547 (94.6)	555 (87.7)	419 (68.0)	606(88.0)	453(93.8)	3683(89.2)
Healthcare student	18 (3.5)	1 (0.2)	9 (1.6)	22 (3.5)	37 (6.0)	25(3.6)	12(2.5)	124(3.0)
Healthcare worker	7 (1.4)	3 (0.5)	22 (3.8)	56 (8.8)	160 (26.0)	58(8.4)	18(3.7)	324(7.8)
**Income category *n* (%)**								
Low	4 (0.8)	73 (11.8)	281 (48.6)	240 (37.9)	61 (9.9)	56(8.1)	35(7.2)	750(18.2)
Lower & middle	486 (95.1)	535 (86.2)	237 (41.0)	288 (45.5)	386 (62.7)	589(85.5)	328(67.9)	2849(69.0)
High/Upper middle	21 (4.1)	13 (2.1)	60 (10.4)	105 (16.6)	169 (27.4)	44(6.4)	120(24.8)	532(12.9)
Chronic disease *n* (%) *								
No	434 (84.9)	603 (97.1)	441 (76.3)	462 (73.0)	510 (82.8)	636(92.3)	443(91.7)	3529(85.4)
Yes	77 (15.1)	18 (2.9)	137 (23.7)	171 (27.0)	106 (17.2)	53(7.7)	40(8.3)	602(14.6)

*: Hypertension, diabetes, HIV/AIDS, cancer.

**Table 2:** COVID-19 vaccine acceptance and beliefs on COVID-19 existence by provinces.

Characteristics	Province							*p*-Value
	Haut-Katanga	Kasaï-Central	Kasaï-Oriental	Kinshasa	Kongo-Central	Kwilu	Nord-Kivu	
**Vaccine Acceptance *n* (%)**								
Yes	184 (36)	583 (94	489 (85)	262 (41)	296 (48)	269 (39)	227 (47)	
No	327 (64)	38 (6)	89 (15)	371 (59)	320 (52)	420 (61)	256 (53)	<0.001
**Believe COVID-19 Exists *n* (%)**								
No	69 (13.5)	16 (2.6)	544 (94.1)	72 (11.4)	78 (12.7)	198 (28.7)	19 (3.9)	
Yes	442 (86.5)	605 (97.4)	34 (5.9)	561 (88.6)	538 (87.3)	491 (71.3)	464 (96.1)	<0.001
Total	511 (12.4)	621 (15.0)	578 (14.0)	633 (15.3)	616 (14.9)	689 (16.7)	483 (11.7)	

**Table 3:** Factors associated with COVID vaccine acceptance in the DRC.

Factor	Crude OR (CI 95%)	*p*-Value	Adjusted OR (95% CI)	*p*-Value
Gender				
Male	1			
Female	1.00 (0.86–1.16)	0.99		
Income Category				
Low	1			
Lower Middle	2.31 (1.85–2.88)	<0.001	1.85 (1.46–2.35)	<0.001
High/Upper	4.83 (3.66–6.34)		2.91 (2.15–3.93)	
Healthcare worker				
No	1			
Yes	0.35 (0.28–0.44)	<0.001	0.46 (0.36–0.58)	<0.001
Education				
Secondary and Primary	1			
University	1.82 (1.55–2.13)	<0.001		
Marital status				
Single/Separated	1			
Married/union	1.12 (0.96–1.32)	0.15		
Religion				
None	1			
Catholic	1.54 (1.03–2.30)			
Protestant/Adventist	1.63 (1.08–2.45)	0.012		
Pentecostal/Revival	1.78 (1.15–2.76)			
Other	1.27 (0.85–1.91)			
COVID Testing				
Not Tested	1			
Tested	6.040 (4.70–7.76)	<0.001	4.71 (3.62–6.12)	<0.001
Believe COVID Exists				
No	1			
Yes	7.78 (5.75–10.53)	<0.001	6.04 (4.42–8.23)	<0.001
Province-Specific Level of Acceptance				
Low Acceptance	1			
High Acceptance	6.14 (1.94–19.40)	0.002	14.45 (2.91–71.65)	0.001
Province-Specific Level of Perception				
Low Perception	1			
High Perception	0.73 (0.11–5.02)	0.75		
Chronic Disease				
No	1			
Yes	1.26 (1.04–1.53)	0.017		
				

## Data Availability

Data are available upon reasonable request. Data are available on the International Consortium (International Citizen Project COVID-19 (ICPcovid): http://www.icpcovid.com) website and could be used by other investigators on request. De-identified participant data are available. My ORCID identifier is 0000-0002-3022-4879 and my email is: jditekemenaotmail.fr.

## Supplementary Materials

The following are available online at https://www.mdpi.com/2076-393X/9/2/153/s1.

## Appendix A

**Table A1:** Province level variance of the multilevel logistic models predicting acceptance of vaccine in DRC.

Random Effect	Model 1	Model 2	Model 3 (Final)
Community level variance	1.39	2.87	1.09
ICC (%)	29.7	46.6	24.9
PCV (%)	Reference		21.6
Model fitness statistics (AIC)	4798	4260	4257

ICC intra-class correlation, PCV proportional change in variance, AIC Akaike Information Criteria, CI confidence interval.

### Random Effect Results

The null model has an ICC value of 29.7%, indicating that about 30% of the total variation on vaccine acceptance occurred at the community or province level and is attributable to the province-level factors. After considering both individual and province-level covariates, province-level variability was reduced to 24.9%. The model also showed that 21.6% of province-level variance on vaccine acceptance was explained by the combined factors at both the individual and province levels.

**Table A2:** Reasons for vaccine hesitancy among healthcare workers in the DRC.

Type of MCM	Frequency (*n*)	Percentage (%)
I don’t trust it, *n* (%)	89	59.3
Prefer not to answer, *n* (%)	31	20.6
They want to kill us, *n* (%)	10	6.6
They want to make us sterile, *n* (%)	5	3.3
Other reasons, *n* (%)	14	9.3
Fear of serving once again as a “lab mouse” as for my Ebola vaccination	1	7.1
We had few deaths in my country, we can solve this in another way	1	7.1
Being afraid	1	7.1
It should be first a vaccination at a global scale	1	7.1
I have no details about the existence of the virus, so can’t take the vaccine	1	7.1
Vaccine must be tested in white people first before coming to us	1	7.1
My religion forbids me any kind of vaccine	2	14.3
I don’t know yet, I will decide when that moment will come	3	21.4
There are several conspiracy theories going around	1	7.1
External vaccines have nanoparticles that I don’t want	1	7.1
I’m immune-deficient	1	7.1
Total	149	100
