# Supplementary material for: COVID-19 Vaccine Acceptance in the Democratic Republic of Congo: A Cross-Sectional Survey
Source: Vaccines (Basel). 2021 Feb 14;9(2):153. doi: 10.3390/vaccines9020153 (PMC7917589; doi:10.3390/vaccines9020153)
Supplement: Supplementary file 1 [file vaccines-09-00153-s001.pdf]

DRC Covid-19 Questionnaire [ROUND 2b]

Socio-demographic information

Introduction
The coronavirus (also called Covid-19) is a virus that infects individuals around the world and can cause respiratory problems and in some cases, death. The Covid-19 is already circulating in DRC, and a lot of efforts are being invested to prevent its spread in our community. In that light, the Congolese government took a number of measures during the last couple of weeks to prevent the spread of Covid-19. People were asked to stay home, to avoid gatherings and travelings, and to frequently wash their hands. With this short questionnaire, we try to find out how you experience and apply the instructions implemented against coronavirus. We call upon volunteers to complete this questionnaire as truthfully as possible; because this helps us investigate which measures are effective in these exceptional times. It should take you about 10 minutes to answer the questions. This is not an exam and your name is not required, so please give accurate answers.

Participation in the survey is voluntary, you can cancel it at any time without any disadvantages. No names or sensitive information will be required from you. The information obtained will be stored anonymously and treated confidentially. It will be used to understand the feasibility and effectiveness of implementing preventive measures for the coronavirus at the individual and country levels. There is no risk associated with this survey, as we will not meet you in person and no names, contact information or biological samples will be obtained from you. Moreover, the survey is hosted in a secure website in Belgium. If you would like to know more about the researchers involved in this study and our privacy policy, you can find this on the www.ICPCovid.com website.

Q: I fully understand what this study is about, and I freely consent to participate. All the information I provide can be used by researchers to better understand coronavirus disease in Congo. (*)

Type: checkbox

A: checkbox

Q: Age (*)

Type: number

A: number (min: 1 / max: 110 / step: 1)

Q: Sex (*)

Type: choice

A: one of the following:

male	=>	*Male*
female	=>	*Female*

Q: Nationality (*)

Type: choice

A: one of the following:

local	=>	*Congolese*
foreigner	=>	*Foreigner*

Q: In which province of Congo do you live? (*)

Type: choice

A: one of the following:

bas_uele	=>	*Bas-Uele*
equateur	=>	*Équateur*
haut_katanga	=>	*Haut-Katanga*
haut_lomami	=>	*Haut-Lomami*
haut-uele	=>	*Haut-Uele*
ituri	=>	*Ituri*
kasaï	=>	*Kasaï*
kasaï_central	=>	*Kasaï-Central*
kasaï_oriental	=>	*Kasaï-Oriental*
kinshasa	=>	*Kinshasa*
kongo_central	=>	*Kongo-Central*
kwango	=>	*Kwango*
kwilu	=>	*Kwilu*
lomami	=>	*Lomami*
lualaba	=>	*Lualaba*
mai_ndombe	=>	*Mai-Ndombe*
maniema	=>	*Maniema*
mongala	=>	*Mongala*
nord_kivu	=>	*Nord-Kivu*
nord_ubangi	=>	*Nord-Ubangi*
sankuru	=>	*Sankuru*
sud_kivu	=>	*Sud-Kivu*
sud_ubangi	=>	*Sud-Ubangi*
tanganyika	=>	*Tanganyika*
tshopo	=>	*Tshopo*
tshuapa	=>	*Tshuapa*

Q: Religion (*)

Type: choice

A: one of the following:

protestant	=>	*Protestant*
catholic	=>	*Catholic*
pentecostal	=>	*Pentecostal*
adventist	=>	*Seventh Day Adventist*
muslim	=>	*Muslim*
jehovah_witness	=>	*Jehovah Witness*
other	=>	*Other*
none	=>	*None*

Q: Highest educational level (*)

Type: choice

A: one of the following:

primary	=>	*Primary*
Secondary	=>	*Secondary*
University Undergraduate degree holder	=>	*University Undergraduate degree holder*
University Postgraduate degree holder	=>	*University Postgraduate degree holder*

Q: Marital status (*)

Type: choice

A: one of the following:

single	=>	*Single*
legally_married	=>	*Legally married*
cohabitation	=>	*Cohabitation*
divorced	=>	*Divorced*
widow_widower	=>	*Widow/widower*

Q: Do you live in: (*)

Type: choice

A: one of the following:

rural_area	=>	*a rural area/village?*
suburb	=>	*a suburban area?*
urban_town	=>	*an urban area/town*
other	=>	*Other (specify)*

Q: Specify urban area: (*)

Type: choice

A: one of the following:

headquarter_region	=>	*In a regional headquarter*
capital_country	=>	*In the capital of the country*
economic_capital	=>	*In the economic capital*
big_city	=>	*In another big city*

Visible if

Q:	A:
Do you live in:	- value => urban_town

Q: Specify where you live: (*)

Type: text

A: text input

Visible if

Q:	A:
Do you live in:	- value => other

Q: What are your housing conditions? (*)

Type: choice

A: one of the following:

room	=>	*a room*
studio	=>	*a studio (a room, with kitchen and toilet)*
apartment	=>	*an apartment with many other households in the same building*
villa	=>	*a villa*
hut	=>	*a hut*
homeless	=>	*homeless*

Q: In the context of your country, what category is most appropriate for your current socio-economic situation? (*)

Type: choice

A: one of the following:

low_income	=>	*Low income category*
low_middle	=>	*Lower middle income category*
upper_middle	=>	*Upper middle income category*
high_income	=>	*High income category*

Q: Is this questionnaire administered to you by a research officer? (*)

Type: choice

A: one of the following:

yes	=>	*Yes, a researcher is noting down my responses*
no	=>	*No, I am answering the questions myself on the website*

Q: If yes, please write the Researcher's code: (*)

Type: text

A: text input

Visible if

Q:	A:
Is this questionnaire administered to you by a research officer?	- value => yes

Daily life during the coronavirus epidemic

Q: How do you obtain information about the coronavirus epidemic? (many answers possible) (*)

Type: choice_multiple

A: multiple answers possible:

family_neighbours	=>	*From family, neighbours or friends*
radio	=>	*From the radio*
television	=>	*From the television*
social_media	=>	*From the social media (WhatsApp, Facebook, Twitter, etc)*
government	=>	*From government authorities*
chief	=>	*From the village/quarter chief*
religious	=>	*From the religious authorities*
health_personnel	=>	*From healthcare workers (nurses, doctors, etc)*

Q: How many people apart from your housemates did you talk to yesterday face to face (not by phone, chat etc)?(*)

Type: number

A: number (min: n/a / max: n/a / step: n/a)

Q: When was the last time you shook hands, hugged, gave a kiss or had any form of physical contact with someone other than a housemate, during the last month? (*)

Type: choice

A: one of the following:

today	=>	*Today*
last_two_days	=>	*Last two days*
last_3_to_6_days	=>	*Last 3 to 6 days*
one_to_two_weeks	=>	*Between one to two weeks ago*
more_than_two_weeks	=>	*More than two weeks ago*
no_contacts	=>	*No contacts with persons outside my household during the last month*

Q: During the last week did you have difficulties in obtaining food or essential needs??(*)

Type: choice

A: one of the following:

Yes	=>	*Yes*
No	=>	*No*

Q: What was the most important reason you had difficulties in obtaining food last week?(*)

Type: choice

A: one of the following:

no_money	=>	*No money due to lost revenue since the coronavirus epidemic started*
little_food_available	=>	*Little food available in shops/market*
expensive	=>	*Food has become too expensive since the coronavirus epidemic started*

unsafe_to_go_out	=>	*I felt it was unsafe to go out to buy food*
to_ill_to_go_out	=>	*I was too ill to go out*

Visible if

Q:	A:
During the last week did you have difficulties in obtaining food or essential needs??	- value => Yes

Q: During the last week, how worried or afraid were you about your health? **(*)**

Type: choice_scale

A: 1 = not worried to 5 = extremely worried

1	=>	*1*
2	=>	*2*
3	=>	*3*
4	=>	*4*
5	=>	*5*

Q: Have you suffered any form of violence or discrimination because of the measures taken against the coronavirus? (many answers possible) **(*)**

Type: choice_multiple

A: multiple answers possible:

home_violence	=>	*Physical violence at home*
outside_violence	=>	*Physical violence outside*
socio_economic_discrimination	=>	*Discrimination because of my social/economic status*
quarantined	=>	*Discrimination because I was quarantined*
ethnic_discrimination	=>	*Discrimination because of my ethnicity*
no_violence_discrimination	=>	*No violence or discrimination*

Q: During the COVID-19 lockdown, have you experienced violence from your sexual partner (if applicable)? **(*)**

Type: choice

A: one of the following:

yes	=>	*Yes*
no	=>	*No*
not_applicable	=>	*Not applicable*

Q: If yes, which kind of violence did you experience? **(*)**

Type: choice

A: one of the following:

verbal	=>	*Verbal violence*
physical	=>	*Physical violence*

sexual	=>	*Sexual violence, including rape*
other	=>	*Other*

Visible if

Q:	A:
During the COVID-19 lockdown, have you experienced violence from your sexual partner (if applicable)?	- value => yes

Q: During the COVID-19 lockdown period, did any woman in your household become pregnant? (*)

Type: choice

A: one of the following:

yes_under20	=>	*Yes, a woman younger than 20 years became pregnant*
yes_over20	=>	*Yes, a woman aged at least 20 years became pregnant*
no	=>	*No pregnancy occurred in my household*
dont_know	=>	*I don't know*

Professional life during the coronavirus epidemic

Q: What do you do for a living? (*)

Type: choice

A: one of the following:

student	=>	*Student*
jobless	=>	*Jobless*
self_employed	=>	*Self-employed*
company	=>	*Work for a person, institution or company*
government	=>	*Work for the government*

Q: Are you a healthcare worker or a student working in the health sector? (*)

Type: choice

A: one of the following:

yes_worker	=>	*Yes, I am a healthcare worker*
yes_student	=>	*Yes, I am a healthcare student*
no	=>	*No*

Q: If yes, what is your main function in the health structure? (*)

Type: choice

A: one of the following:

nurse	=>	*Nurse*
gp	=>	*Medical doctor: general practitioner*

specialist	=>	*Medical doctor: resident/specialist*
lab	=>	*Laboratory staff*
pharmacy	=>	*Pharmacy staff*
research_maintenance	=>	*Research/Maintenance*
admin_finance	=>	*Administration/finance*
technician	=>	*Technician (imaging, specialized services, etc)*

Visible if

Q:	A:
0	- :input[name="are_you_a_healthcare_worker_or_a_student_working_in_the_health_s"] =>
1	
2	- :input[name="are_you_a_healthcare_worker_or_a_student_working_in_the_health_s"] =>

Q: Have you been given any protective equipment at your place of work? (many answers possible)(*)

Type: choice_multiple

A: multiple answers possible:

masks	=>	*Face masks*
gloves	=>	*Gloves*
shield	=>	*Face shield*
boots	=>	*Boots*
apron	=>	*Full protective apron*
gel	=>	*Hand gels/sanitizers*
none	=>	*None*

Visible if

Q:	A:
0	- :input[name="are_you_a_healthcare_worker_or_a_student_working_in_the_health_s"] =>
1	
2	- :input[name="are_you_a_healthcare_worker_or_a_student_working_in_the_health_s"] =>

Q: What are your current working conditions? (*)

Type: choice

A: one of the following:

worker_from_home	=>	*Worker from home*
worker_open_space	=>	*Worker in an open space (market, shop, roadside, etc)*
worker_closed_space_alone	=>	*Worker in a closed indoor space alone (office, …., etc.)*
worker_closed_space_people	=>	*Worker in a closed indoor space with several people (office, …., etc.)*
not_applicable	=>	*Not applicable (if jobless or student)*

Q: How many days per week do you usually go to work? (*)

Type: number

A: number (min: n/a / max: 7 / step: n/a)

Q: How many days did you (physically) go to work last week? (*)

Type: number

A: number (min: n/a / max: 7 / step: n/a)

Q: Are you exclusively working from home this week? (*)

Type: choice

A: one of the following:

yes	=>	*Yes*
no	=>	*No*
not_applicable	=>	*Not applicable (if jobless or student)*

Q: Why are you not working from home? (*)

Type: choice

A: one of the following:

not_possible	=>	*It is not possible with my job*
not_allowed	=>	*It is possible, but is not allowed by my employer*
home_not_working	=>	*I am at home but not working*
no_risk_to_go_out	=>	*I don't think there is any risk to go out*
other	=>	*Other*

Visible if

Q:	A:
Are you exclusively working from home this week?	- value => no

Q: What transportation means did you use to go to work? (*)

Type: choice

A: one of the following:

public_transport	=>	*By public transport with multiple people (taxi, bus, motorcycle, etc)*
hired_vehicle	=>	*Hired a vehicle for myself and/or family members (private taxi, rented car, etc)*
own_transport	=>	*personal vehicle (Car, motorcycle, etc)*
walked	=>	*Walked to work*

Visible if

Q:	A:
Are you exclusively working from home this week?	- value => no

Personal preventive measures for coronavirus

Q: Have you had a mask during the past 7 days? (*)

Type: choice

A: one of the following:

yes	=>	*Yes*
no	=>	*No*

Q: If yes, how did you get a mask? (*)

Type: choice

A: one of the following:

bought	=>	*I bought it*
given	=>	*It was given to me at work*
someone	=>	*Someone gave it to me*
healthcare	=>	*As a healthcare worker, I was given one at work*

Visible if

Q:	A:
Have you had a mask during the past 7 days?	- value => yes

Q: Have you worn a mask during the past 7 days? (*)

Type: choice

A: one of the following:

yes	=>	*Yes*
no	=>	*No*

Q: At this moment that you are filling this questionnaire, are you wearing a mask? (*)

Type: choice

A: one of the following:

yes	=>	*Yes*
no	=>	*No*

Q: If yes, please select one option below: (*)

Type: choice

A: one of the following:

nose_mouth	=>	*The mask covers my mouth and nose*
only_mouth	=>	*The mask covers only my mouth, but not my nose*
under_chin	=>	*The mask is under my chin, not covering my nose or my mouth*

Visible if

Q:	A:
At this moment that you are filling this questionnaire, are you wearing a mask?	- value => yes

Q: I wear a face mask when going outside (*)

Type: choice

A: one of the following:

yes	=>	*Yes*
no	=>	*No*
na	=>	*Not applicable (I do not go out)*

Q: If yes, when/where do you wear a face mask? (many answers possible) (*)

Type: choice_multiple

A: multiple answers possible:

home	=>	*At home*
work	=>	*At work*
sometimes_out	=>	*Sometimes when I go out*
always_out	=>	*Everytime I go out*

Visible if

Q:	A:
I wear a face mask when going outside	- value => yes

Q: If no, what is (are) the reason(s) for not using face masks? (many answers possible) (*)

Type: choice_multiple

A: multiple answers possible:

no_mask	=>	*I do not have a mask*
no_money	=>	*I don't have money to buy masks*
where_to_get	=>	*I don't know where to get a mask*
not_needed	=>	*I don't think that masks are needed*

Visible if

Q:	A:
I wear a face mask when going outside	- value => no

Q: (OPTIONAL) Please write down any comments about face mask use:

Type: text

A: text input

Q: I follow the social 1.5-2m meters distance rule (*)

Type: choice

A: one of the following:

Yes	=>	*Yes*
No	=>	*No*

Q: When I cough or sneeze, I do so in my elbow and/or I cover my mouth with a tissue paper(*)

Type: choice

A: one of the following:

Yes	=>	*Yes*
No	=>	*No*

Q: When I cough or sneeze, I usually wash/desinfect my hands immediately afterwards (*)

Type: choice

A: one of the following:

Yes	=>	*Yes*
No	=>	*No*

Q: I wash my hands using soap and water regularly during the day (*)

Type: choice

A: one of the following:

Yes	=>	*Yes*
No	=>	*No*

Q: I use a hand sanitizer regularly during the day (*)

Type: choice

A: one of the following:

Yes	=>	*Yes*
No	=>	*No*

Q: What constraints to hand washing/use of hand sanitizers do you face?

Type: text

A: text input

Visible if

Q:	A:
I wash my hands using soap and water regularly during the day	- value => No
I use a hand sanitizer regularly during the day	- value => No

Q: I avoid touching my face (eyes, nose and mouth) (*)

Type: choice

A: one of the following:

Yes	=>	*Yes*
No	=>	*No*

Q: I disinfect my phone whenever I return home (*)

Type: choice

A: one of the following:

yes	=>	*Yes*
no	=>	*No*
na	=>	*Not applicable*

Q: I stay home when I feel flu-like symptoms (*)

Type: choice

A: one of the following:

Yes	=>	*Yes*
No	=>	*No*

Q: On a scale of 1 to 5, how difficult is it for you personally to follow the protective measure of staying home as much as possible? (*)

Type: choice_scale

A: 1 = not difficult at all; 5 = extremely difficult

1	=>	*1*
2	=>	*2*
3	=>	*3*
4	=>	*4*
5	=>	*5*

Community preventive measures for coronavirus

Q: Were you in a meeting or gathering with with 50 persons or more during the last 7 days?(*)

Type: choice

A: one of the following:

Yes	=>	*Yes*
No	=>	*No*

Q: What was the nature of the gathering? (multiple answers possible) (*)

Type: choice_multiple

A: multiple answers possible:

restaurant	=>	*Restaurant*
bar	=>	*Bar*
club	=>	*Club*
party	=>	*Party*
funeral	=>	*Funeral*
religious	=>	*Religious gathering*
family	=>	*Family gathering*
sport	=>	*Sports event*
other	=>	*Other*

Visible if

Q:	A:
Were you in a meeting or gathering with with 50 persons or more during the last 7 days?	- value => Yes

Q: Were you in a car with 4 or more other persons during the last 7 days?(*)

Type: choice

A: one of the following:

Yes	=>	*Yes*
No	=>	*No*

Q: Were you in a public gym in the past 7 days ? (*)

Type: choice

A: one of the following:

Yes	=>	*Yes*
No	=>	*No*

Q: Did you go to a barber/hairdresser, beauty parlour, massages, spa, or nail studio in the past 7 days ?(*)

Type: choice

A: one of the following:

Yes	=>	*Yes*
No	=>	*No*

Q: Did you go to a market in the past 7 days ? (*)

Type: choice

A: one of the following:

Yes	=>	*Yes*
No	=>	*No*

Q: Did you travel in the past 7 days? (*)

Type: choice

A: one of the following:

yes_other_regions	=>	*Yes, I travelled to other regions of the country*
yes_outside_country	=>	*Yes, I travelled outside of the country*
no_travel	=>	*No travel*

Q: During the last week, how worried or afraid were you about the health of your loved ones?(*)

Type: choice_scale

A: 1 = not worried to 5 = extremely worried

1	=>	*1*
2	=>	*2*
3	=>	*3*
4	=>	*4*
5	=>	*5*

Q: On a scale of 1 to 10, can you indicate the extent to which people in your environment have practically adapted their behavior to the government recommendations? (*)

Type: choice_scale

A: 1 = no adaptions, 10 = very strong adaptation

1	=>	*1*
2	=>	*2*
3	=>	*3*
4	=>	*4*
5	=>	*5*
6	=>	*6*
7	=>	*7*
8	=>	*8*
9	=>	*9*
10	=>	*10*

Questions related to your personal health

Q: Have you been eating more healthy food such as fruits and vegetables since the coronavirus epidemic started?(*)

Type: choice

A: one of the following:

Yes	=>	*Yes*

No	=>	*No*

Q: Have you been taking more vitamin tablets since the coronavirus epidemic started? (*)

Type: choice

A: one of the following:

Yes	=>	*Yes*
No	=>	*No*

Q: Did you experience any of the following flu-like symptoms during the last 14 days? (multiple options possible)(*)

Type: choice_multiple

A: multiple answers possible:

fever	=>	*Fever*
headaches	=>	*Headaches*
sore_throat	=>	*Sore throat*
loss_taste	=>	*Loss of taste*
loss_smell	=>	*Loss of smell*
stuffy_nose	=>	*Stuffy and/or running nose*
dry_cough	=>	*Dry cough*
productive_cough	=>	*Productive cough*
shortness_breath	=>	*Shortness of breath*
muscle_pain	=>	*Muscle or body pains*
weakness	=>	*General weakness*
nausea	=>	*Nausea*
diarrhea	=>	*Diarrhea*
none	=>	*None of the above*

Q: For how many days did you have flu-like symptoms? (*)

Type: number

A: number (min: 1 / max: 50 / step: n/a)

Visible if

Q:	A:
:input[name="if_yes_which_symptoms_did_you_experience_multiple_options_possib[none]"]	- unchecked => 1

Q: Have any of your housemates had flu-like symptoms in the last 14 days? (*)

Type: choice

A: one of the following:

yes	=>	*Yes*
no	=>	*No*

do_not_know	=>	*Do not know*

Q: Have you been tested for the coronavirus? (*)

Type: choice

A: one of the following:

yes	=>	*Yes*
no	=>	*No*

Q: If yes, what was the result of the test? (*)

Type: choice

A: one of the following:

positive	=>	*Positive*
negative	=>	*Negative*
do_not_know	=>	*Do not know yet*

Visible if

Q:	A:
Have you been tested for the coronavirus?	- value => yes

Q: When was your coronavirus test done? (*)

Type: choice

A: one of the following:

two_weeks	=>	*During the past two weeks*
one_month	=>	*Between two weeks and one month*
more_than_one_month	=>	*More than one month ago*

Visible if

Q:	A:
Have you been tested for the coronavirus?	- value => yes

Q: Do you know anyone who has suffered from COVID-19? (many options possible) (*)

Type: choice_multiple

A: multiple answers possible:

friend	=>	*Yes, a friend*
relative	=>	*Yes, a relative*
neighbour	=>	*Yes, someone in my neighbourhood*
other	=>	*Yes, other persons*
no	=>	*No, I don't know anyone who has suffered from COVID-19*

Q: Do you know anyone who has died of COVID-19? (many options possible)(*)

Type: choice_multiple

A: multiple answers possible:

friend	=>	*Yes, a friend*
relative	=>	*Yes, a relative*
neighbour	=>	*Yes, someone in my neighbourhood*
other	=>	*Yes, other persons*
no	=>	*No, I don't know anyone who has suffered from COVID-19*

Q: Do you smoke? (*)

Type: choice

A: one of the following:

Yes	=>	*Yes*
No	=>	*No*

Q: Do you have any of the following chronic/underlying disease? (many answers possible)(*)

Type: choice_multiple

A: multiple answers possible:

heart_disease	=>	*Heart disease*
hypertension	=>	*Hypertension*
diabetes	=>	*Diabetes*
cancer	=>	*Cancer*
hiv	=>	*HIV*
tb	=>	*Tuberculosis*
asthma	=>	*Asthma*
other	=>	*Other*
none	=>	*None of the above*

Q: If you have an underlying disease did you experience difficulties to obtain your medication since the COVID outbreak started? (*)

Type: choice

A: one of the following:

Yes	=>	*Yes*
No	=>	*No*

Visible if

Q:	A:
:input[name="do_you_have_any_of_the_following_underlying_disease_many_answers[none]"]	- unchecked => 1

Q: Do you think that the coronavirus really exists in your province? (*)

Type: choice

A: one of the following:

yes	=>	*Yes, it exists*
no	=>	*No, it is a lie*

Q: If no, why do you think that it is a lie? (*)

Type: text

A: text input

Visible if

Q:	A:
Do you think that the coronavirus really exists in your province?	- value => no

Q: Do you think COVID-19 is treatable? (*)

Type: choice

A: one of the following:

yes	=>	*Yes*
no	=>	*No*
NA	=>	*Not applicable (I don't believe that coronavirus exists)*

Q: In your opinion, was the coronavirus lockdown necessary in Congo? (*)

Type: choice

A: one of the following:

yes	=>	*Yes*
no	=>	*No*

Q: Would you consent to receive a COVID-19 vaccine if it becomes available in our country? (*)

Type: choice

A: one of the following:

yes	=>	*Yes*
no	=>	*No*

Q: If no, why would you refuse the vaccine? (*)

Type: choice

A: one of the following:

no_trust	=>	*I do not trust it*
kill	=>	*They want to kill us*

sterile	=>	*The want to make us sterile*
na	=>	*Prefer not to answer*
other	=>	*Other reason (specify)*

Visible if

Q:	A:
Would you consent to receive a COVID-19 vaccine if it becomes available in our country?	- value => no

Q: Please specify other raison:

Type: text

A: text input

Visible if

Q:	A:
If no, why would you refuse the vaccine?	- value => other
